# Analysis of combining ability and stability of yield characteristics of upland cotton in two years

**DOI:** 10.7717/peerj.19716

**Published:** 2025-07-30

**Authors:** Xiaoman Ma, Weifeng Guo, Liangrong He, Xinchuan Cao

**Affiliations:** 1 College of Agriculture, Tarim University, Alar, China; 2 Key Laboratory of Genetic Improvement and Efficient Production for Specialty Crops in Arid Southern Xinjiang of Xinjiang Corps, Alar, China

**Keywords:** Land cotton, Complete biallelic hybridisation, Cooperativeness, Stability

## Abstract

**Objective:**

Combining ability analysis forms the basis for selecting suitable parents and hybrid combinations. The performance of combining ability is influenced significantly by environmental conditions. Therefore, it is essential to comprehensively evaluate advantageous parents and hybrid combinations through multi-year experiments.

**Methods:**

In this study, seven parental lines were crossed using a complete diallel design [p(p−1)/2], producing 21 combinations. Yield-related traits, including boll number per plant, seed cotton per plant, lint cotton per plant, boll weight, lint percentage, and seed index, were measured in the parental lines and their hybrid F1 generations over two consecutive years. Combining ability and interannual stability were subsequently analysed.

**Results:**

Cotton yield traits were significantly influenced by environmental factors such as weather, temperature, and soil conditions. Interannual variation and genotype-year interactions contributed substantially to variation in yield traits. The interactions between year and both general combining ability (GCA) and specific combining ability (SCA) were significant or highly significant, with GCA stability being relatively low. Yield traits were governed by both additive and dominance genetic effects, with additive effects being predominant. The comparison coefficients for stability of GCA for lint cotton per plant and seed cotton per plant across years were relatively high (51.372% and 55.187%, respectively), whereas SCA stability coefficients for lint percentage and boll weight were comparatively lower (44.986% and 48.645%, respectively). Parent Xinluzao62 (Parent 7) exhibited both high and stable GCA and can therefore be recommended as a backbone parent for yield improvement. Additionally, eight hybrid F1 combinations showing excellent and stable SCA were identified, suitable for use as dominant combinations.

**Conclusion:**

The GCA and SCA for yield traits in upland cotton are notably influenced by environmental factors such as climate and soil. Yield performance varies considerably across different years and locations. Therefore, selections based on multi-year and multi-location trials provide more reliable results, offering a solid theoretical basis for developing high-yield cotton varieties.

## Introduction

Xinjiang possesses significant advantages for cotton cultivation owing to its distinctive climate and geographical conditions. It is currently China’s largest cotton production base (Luo, 2021; Zhang, Chen & Zhao, 2023). Crossbreeding remains a primary method in cotton breeding, with combining ability analysis being an essential approach for selecting suitable hybrid parents and combinations (Amein et al., 2013; Al-Hibbiny, 2015). Combining ability analysis is extensively employed in diverse crops, including maize (Mishra et al., 2024; Subba et al., 2022), chili (Siddappa, Ravindra & Shashikanth, 2019; Savitha, Pugalendhi & Natarajan, 2013), beans (Yang et al., 2023; Saba et al., 2020; Han et al., 2018), rice (Sujeet Kumar & Singh, 2018; Deshmukh, Koshta & Sharma, 2016), and cotton (Li et al., 2024; Patil et al., 2012). Such analysis plays a pivotal role in hybrid breeding and exploiting heterosis. Additionally, GGE biplots—a graphical method that helps elucidate genotype-environment interactions—are widely utilised in various crops to evaluate the stability of yield-related traits (Iqbal et al., 2022; Sadabadi et al., 2018; Ma, Liu & Ye, 2024; Li et al., 2023; Chandra et al., 2022; Mullualem et al., 2024). Environmental conditions, such as drought and salinity, significantly influence cotton yield by decreasing its productivity (Bista et al., 2024).

Most previous studies analyzed data from a single year. However, crop trait compatibility mainly depends on trait phenotypes, which can vary considerably under different environmental conditions, potentially altering combining ability and stability across years. Thus, exploring the interaction between combining ability and annual variations holds substantial importance for future cotton breeding programmes.

Askander (2020) investigated combining ability and stability of wheat using incomplete diallel crosses, reporting significant environmental and hybrid differences. Bhandari et al. (2021) analysed tomato combining abilities under varying environments, concluding that the environment substantially influences all trait-related combining abilities. Tang et al. (2021) performed trials across three locations within one year, highlighting the significant reference value of combining ability and heritability in differing environments. Similarly, Iqbal et al. (2012) examined combining abilities for vegetable beans across three locations within one year, indicating that both general combining ability (GCA) and specific combining ability (SCA) were environmentally influenced. Hao et al. (2005) conducted complete diallel crosses under diverse environments within one year, also concluding that combining ability–environment interactions were prevalent. Habib et al. (2012) analysed yield-trait combining abilities in rice using incomplete diallel crosses and emphasised the importance of both GCA and SCA in trait inheritance.

Trait stability across different environments is also crucial. Typically, additive main effects and multiplicative interaction (AMMI) and GGE biplot methods are employed to analyse genotype–environment interactions. AMMI is a statistical method applied to multi-environment trial data, while GGE biplot provides a graphical statistical analysis of these interactions. Guo et al. (2023) assessed two-year yield data using incomplete diallel crosses, demonstrating the significance of combining ability stability across environments. Rao & Chaturvedi (2024) utilised GGE biplots for stability analysis, identifying high-yield and stable grain genotypes and optimal environments. Premika et al. (2024) suggested that AMMI and GGE biplots effectively assess genotype stability and adaptability across environments. Farwan, Devi & Dhillon (2024) indicated that GGE biplots reveal genotype–environment relationships clearly. Kona et al. (2024) applied both AMMI and GGE biplots to evaluate stability among peanut genotypes. Similarly, Dang et al. (2024) and Wang et al. (2023) demonstrated the efficacy of AMMI and GGE biplots in determining genotype stability and environmental adaptability in sugar beet and other crops, respectively. Nevertheless, these studies primarily used incomplete or complete diallel hybrid combinations across different environments within a single year for various crops. Comprehensive analyses combining variance and stability for complete diallel hybrid combinations across different years in cotton have not yet been reported.

In this study, seven upland cotton varieties (lines) were crossed using a complete diallel method, generating 21 hybrid combinations. Their yield traits, including boll number per plant, seed cotton per plant, leather cotton per plant, boll weight, cotton lint percentage, and seed index, were analysed across two consecutive years. The study aims to identify superior parents and hybrid combinations, providing a theoretical foundation for future breeding and selection of high-yield cotton cultivars.

## Materials & Methods

### Study area overview

The experimental site is located in the southern Tianshan Mountains, Xinjiang Uygur Autonomous Region, China. It is situated at the northern edge of the Taklamakan Desert and the upper reaches of the Tarim River in the 12th Regiment, Alar City, First Division. The site represents a typical artificially developed arid oasis (longitude 80°30′–81°58′E, latitude 40°22′–40°57′N) with a total area of 3,924.32 km^2^ and altitude between 997 and 1,340 m. The climate of the area is classified as warm-temperate continental desert. The soil at the experimental site is sandy loam. The experimental plot length was 10 m, row spacing configuration was (10 cm + 66 cm + 10 cm + 66 cm + 10 cm), and plant spacing was 10.5 cm. Planting was performed using manual broadcasting.

**Figure 1:**
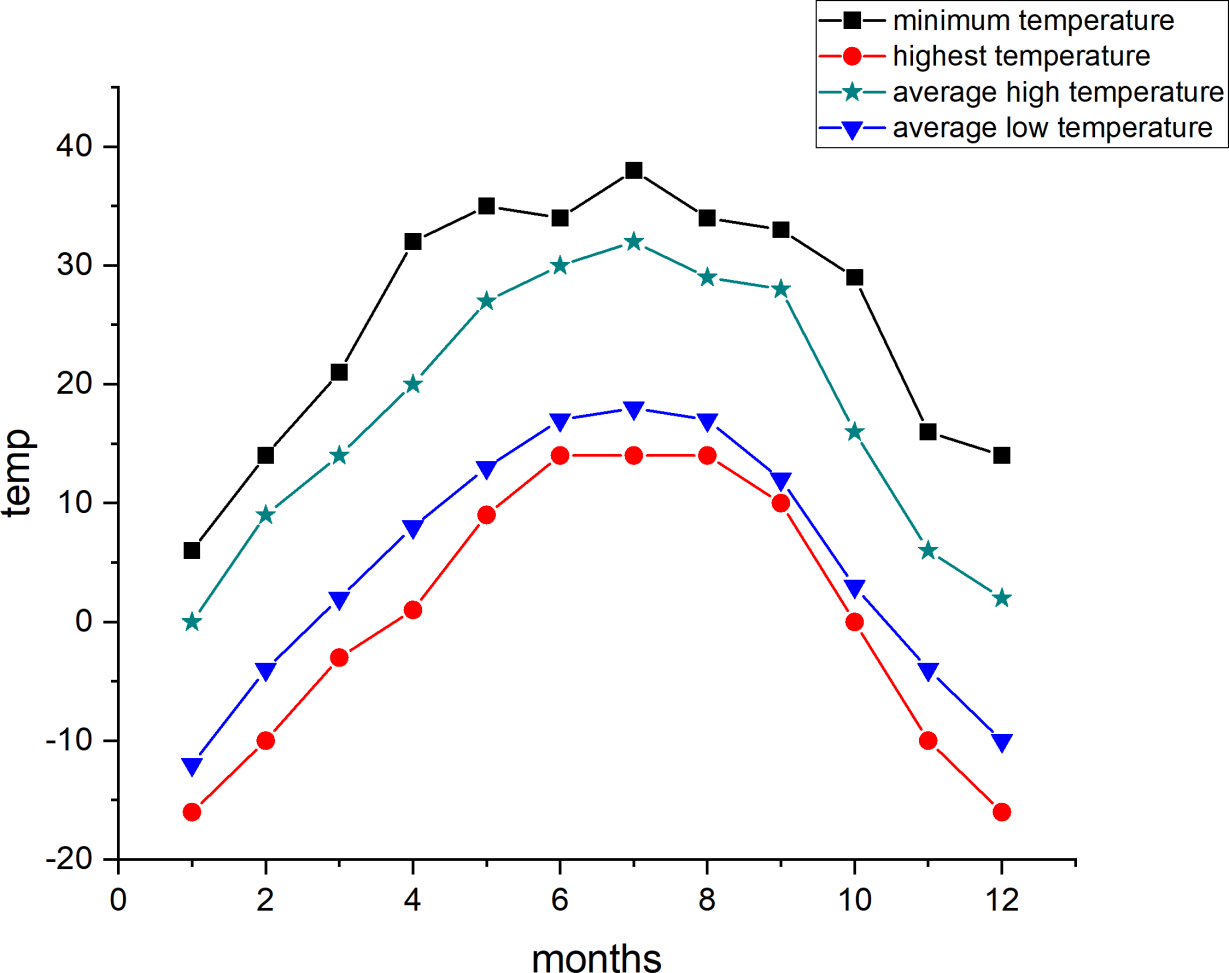
2021 annual climate map.

**Figure 2:**
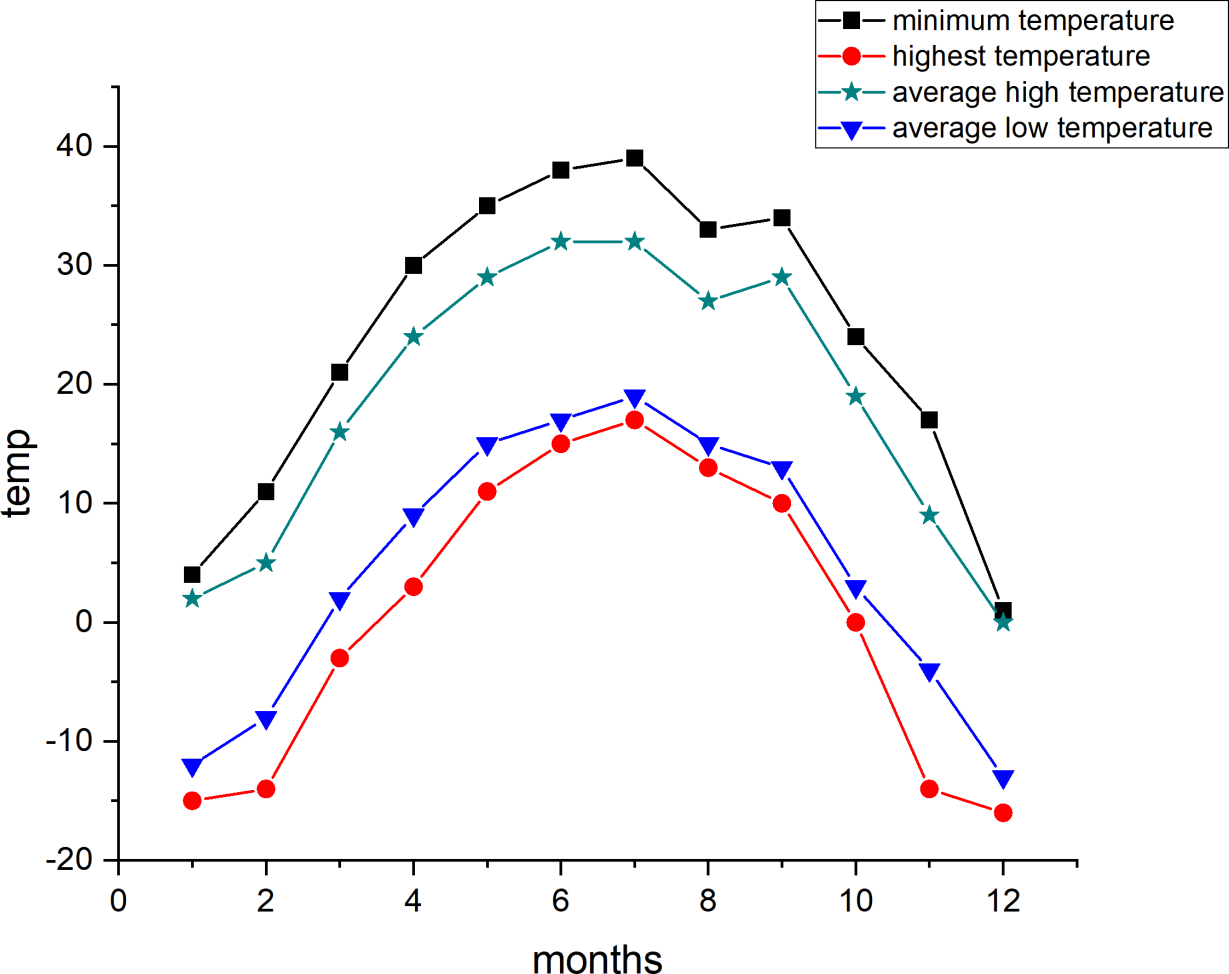
Climate diagram for 2022.

Compared to other regions in Xinjiang Uygur Autonomous Region, Alar City has less snowfall in winter, abundant sunlight and heat resources, significant temperature variations between day and night, and frequent dusty conditions during spring. The average annual temperature is 10.7 °C, with a frost-free period of up to 220 days, making it suitable for cultivating long-staple and fine-staple cotton. Temperature trends for 2021 and 2022 are presented in Figs. 1 and 2, respectively (data sourced from Weather Forecast: https://lishi.tianqi.com/).

### Experimental materials

Seven parental lines—cm3, a115-11, Xinluzhong53, Xinluzhong38, Xinluzhong59, Lu917, and XinLuzao62 (numbered sequentially from 1 to 7)—and their 21 hybrid combinations were used in this study. Hybrid combinations were derived through complete diallel crossing [p(p−1)/2], as detailed in Table 1, provided by the College of Plant Sciences, Tarim University.

**Table 1:** Hybrid combination numbers.

Serial number	Hybrid combination	Serial number	Hybrid combination	Serial number	Hybrid combination
12	cm3 × a115-11	24	a115-11 × Xinluzhong38	37	Xinluzhong 53 × Xinluzao62
13	cm3 × Xinluzhong53	25	a115-11 × Xinluzhong59	45	Xinluzhong 38 × Xinluzhong 59
14	cm3 × Xinluzhong38	26	a115-11 × Lu917	46	Xinluzhong 38 × Lu917
15	cm3 × Xinluzhong59	27	a115-11 × Xinluzao62	47	Xinluzhong 38 × Xinluzao62
16	cm3 × Lu917	34	Xinluzhong53 × Xinluzhong38	56	Xinluzhong 59 × Lu917
17	cm3 × Xinluzao62	35	Xinluzhong53 × Xinluzhong59	57	Xinluzhong 59 × Xinluzao62
23	a115-11 × Xinluzhong53	36	Xinluzhong53 × Lu917	67	Lu917 × Xinluzao62

The seven parental lines and their 21 hybrid combinations were cultivated at the experimental field of the Twelfth Regiment in Aral City in 2021 and 2022. A randomized block design with two replications was employed, using wide-film planting (10 cm + 66 cm + 10 cm), plant spacing of 10.5 cm, and row length of 3 m. Sowing was conducted using mechanical film perforation combined with manual spot sowing, while standard field management practices were uniformly applied.

During the shedding period (mid-October) in both 2021 and 2022, 10 uniformly grown cotton plants from the middle of each plot were selected. Harvested cotton bolls were evaluated in the laboratory for the following traits: boll number per plant (average number of bolls per 10 plants), boll weight (total weight of harvested seed cotton divided by total boll count from 10 plants), seed cotton per plant (total seed cotton harvested divided by 10), leather cotton per plant (average lint cotton after ginning the harvested seed cotton from 10 plants), and seed index (weight of randomly selected 100 cotton seeds).

### Trait determination

1. Plant selection: Ten cotton plants with uniform growth from the centre of each plot were selected to minimize edge effects. Plants with consistent plant height, leaf number, and branch number were ensured.

2. Plant marking: The selected plants were tagged or marked for subsequent identification.

3. Information recording: Each plot’s number and the exact locations of selected plants were documented to guarantee data traceability.

4. Measurement of yield-related traits:

Boll number per plant: Total number of bolls per plant was counted, and the average of ten plants was calculated.

Boll weight: The weight of each cotton boll was measured, and the average boll weight per plant was calculated.

Seed cotton per plant: The total seed cotton weight per plant was calculated (boll number per plant multiplied by boll weight), and the average seed cotton weight per plant for ten plants was determined.

Leather cotton per plant: Lint cotton was obtained by ginning seed cotton using a serrated gin, weighed using an electronic balance, and the average lint cotton per plant for ten plants was calculated.

Seed index: One hundred cotton seeds were randomly selected and weighed to obtain the seed index.

Cotton lint percentage: Calculated as the ratio of leather cotton per plant to seed cotton per plant (cotton lint percentage = leather cotton per plant/seed cotton per plant).

### Data analysis

#### Joint analysis of variance

Joint analysis of variance was employed to analyze repeated experimental data across different years or environmental conditions. Considering multiple factors simultaneously provided a comprehensive analytical perspective.

In this study, data from two years were initially tested for independence (Chi-square test), normality (Shapiro–Wilk test), and homogeneity of variance (Levene test). The PROC GLM program in SAS software was used for joint variance analysis to assess the significance of genotype (G), year (E), and genotype-by-year interactions (G × E). Due to the relatively stable genetic effects of each genotype within the population, sampling was assumed as a fixed model, with year serving as a key variable in the experimental design. Year was set as a fixed effect when evaluating interactions between year and treatment, while grouping within years was typically considered a random effect. Therefore, the mixed-effects model for joint variance analysis was as follows (Guo et al., 2023):

\begin{eqnarray*}{\mathrm{Y}}_{ger}=\mu +{E}_{e}+{G}_{g}+(E\times G)_{eg}+{E}_{r(e)}+{}_{ger} \end{eqnarray*}

where Y_*ger*_ represents the observed value of the g-th genotype, r-th replicate, in the e-th year; µ is the overall experimental mean; *E*_*e*_ is the year effect; *G*_*g*_ is the genotype effect; (*E* × *G*)_*eg*_ is the genotype-year interaction effect; *E*_*r*(*e*)_ is the random block effect within the e-th year; *ɛ*_*ger*_ is the error term, which follows a normal distribution $\mathrm{N}(0,{\delta }_{e}^{2})$).

### Combining ability analysis

Combining ability (GCA and SCA) served as a crucial indicator to evaluate parental and hybrid performance in breeding. The statistical model for observational means obtained from method 2 of diallel hybridization within each environment or year was:

\begin{eqnarray*}{Y}_{ij}=\mu +{g}_{i}+{g}_{j}+{s}_{ij}+{}_{ij}. \end{eqnarray*}

The variety effect is v_ij_ = g_i_ + g_j_ + s_ij_, and there is *s*_*ij*_ = *s*_*ji*_, which means the positive and negative crossing effects are the same. ∑*g*_*i*_ = 0, for each j, ∑*S*_*ij*_ = 0. In the formula, *i*, *j* = 1, 2, 3⋯*p*; when *i* = *j* (parental self crossing): *Y*_*ij*_ = *μ* + 2*g*_*i*_ + *ɛ*_*ij*_; when *i* < *j* (orthogonal combination): *Y*_*ij*_ = *μ* + *g*_*i*_ + *g*_*j*_ + *s*_*ij*_ + *ɛ*_*ij*_.

*μ* is the overall average; *ɛ*_*ij*_ for the error term; it follows the distribution of $N(0,{\delta }_{e}^{2})$).

The combining ability model was defined as follows: *Y*_*ij*_ = *μ* + *GCA*_*i*_ + *GCA*_*j*_ + *SCA*_*ij*_ + *ɛ*_*ij*_.

where µ denotes the overall experimental mean; *GCA*_*i*_ and *GCA*_*j*_ are the GCA effects for parents *i* and *j*, respectively; *SCA*_*ij*_ is the SCA effect between parents *i* and *j*; and *ɛ*_*ij*_ is the error term.

The expression of combining ability was influenced by genotype (parent) as well as environmental factors, such as year, location, and climatic conditions. In this study, DPS19 software (http://www.dpsw.cn/) was used to determine combining abilities. A fixed-effects model was employed to estimate combining ability effects for each trait across the two-year data. Joint variance analysis of GCA and SCA was conducted across years based on the principle of additivity of sums of squares to evaluate the significance and stability of combining abilities across environments.

### Genetic effects analysis

Using the PROC VARCOMP procedure in SAS software, generalized heritability was calculated *via* a mixed linear model (with year as a fixed factor and genotype as a random factor):

\begin{eqnarray*}{h}^{2}= \frac{{\delta }_{G}^{2}}{{\delta }_{G}^{2}+ \frac{{\delta }_{GE}^{2}}{n} + \frac{{\delta }_{}^{2}}{nr} } \times 100\% \end{eqnarray*}

where ${\delta }_{G}^{2}$ is the genotype variance; ${\delta }_{GE}^{2}$ is the genotype-year interaction variance; ${\delta }_{}^{2}$ is the error variance; *n* is the number of years; and *r* is the number of repetitions.

### Correlation and stability analysis of combining ability

Correlations between the combining abilities of various traits across years were calculated to identify traits and combinations less affected by environmental variability. Stability comparison coefficients were calculated separately for each trait. By comparing stability coefficients *C*_*g*_ and *C*_*s*_, parents and hybrids with superior stability were selected to improve breeding efficiency:

\begin{eqnarray*}{C}_{g}& = \frac{{\delta }_{GCA}^{2}}{{\delta }_{GCA}^{2}+{\delta }_{GCA\times E}^{2}} \times 100\% \end{eqnarray*}

\begin{eqnarray*}{C}_{g}& = \frac{{\delta }_{SCA}^{2}}{{\delta }_{SCA}^{2}+{\delta }_{SCA\times E}^{2}} \times 100\% \end{eqnarray*}

where *C*_*g*_ and *C*_*s*_ represent the stability coefficients for GCA and SCA, respectively; ${\delta }_{GCA}^{2}$ and ${\delta }_{GCA\times E}^{2}$ are the variances of general and specific combining abilities; ${\delta }_{SCA}^{2}$ and ${\delta }_{SCA\times E}^{2}$ are the variances of interactions between combining ability and environment.

### Stability analysis of hybrid performance

The AMMI genotype-environment interaction model was applied to evaluate hybrid combinations. GGE biplots displayed performance and stability of combining abilities for each trait across years (Zhao et al., 2024). The AMMI model was defined as follows:

\begin{eqnarray*}{\mathrm{Y}}_{ger}=\mu +{\alpha }_{g}+{\beta }_{e}+\sum _{1}^{n}{\lambda }_{n}{\gamma }_{gn}{\delta }_{en}+{\rho }_{g}+{}_{ger} \end{eqnarray*}

where Y_*ger*_ is the observed value for the g-th genotype in the e-th environment with *r* replicates; *μ* is the overall mean; *α*_*g*_ and *β*_*e*_ denote genotype and environment main effects, respectively; *λ*_*n*_ is the eigenvalue of the n-th principal component; *γ*_*gn*_ and *δ*_*en*_ are genotype and environment scores on the n-th principal component; *ρ*_*g*_ is the residual; *ɛ*_*ger*_ is the experimental error.

Genstat21 software was used for GGE biplot analysis to evaluate combining ability stability and adaptability of parents and hybrid combinations across years.

## Results

### Normality test of residuals for yield traits across years

The residuals of yield traits underwent normality testing (Table 2). Kolmogorov–Smirnov tests indicated Sig. ≥ 0.05, confirming that the experimental data for both years followed a normal distribution.

**Table 2:** Residual normality test of yield traits in different years.

Trait	Year	Kolmogorov–Smirnova		
		Statistic	df	Sig.
Boll number per plant	1	0.085	56	0.200
	2	0.117	56	0.053
Seed cotton per plant	1	0.115	56	0.065
	2	0.093	56	0.200
Leather cotton per plant	1	0.1	56	0.200
	2	0.097	56	0.200
Boll weight	1	0.101	56	0.200
	2	0.092	56	0.200
Cotton lint percentage	1	0.095	56	0.200
	2	0.117	56	0.053
Seed index	1	0.079	56	0.200
	2	0.094	56	0.200

### Joint analysis of variance of yield traits across years

Joint analysis of variance (ANOVA) results (Table 3) revealed no significant differences in seed index and boll weight among blocks within years, while all other traits showed significant or highly significant differences. Yield traits differed significantly or highly significantly across years, except for seed index, suggesting that yields are considerably influenced by environmental factors. Additionally, all yield traits displayed significant or highly significant differences across genotypes (hybrid combinations) and genotype-year interactions. The pervasive significant interactions between genotype and year indicate substantial environmental influences on yield traits.

**Table 3:** Joint ANOVA and mean square significance of genotypes for yield traits in different years.

Nature (*i.e.*, properties of sth)	Group of districts within year	Vintages	Genotypes (hybrid combinations)	Genotype × year	Inaccuracies
Degree of freedom	1	1	27	27	55
Seed index	0.459	0.315	1.377^**^	1.012^**^	0.252
Cotton lint percentage	1.168	527.342^**^	10.929^**^	3.099^**^	0.414^**^
Boll weight	0.224	3.076^**^	0.389^**^	0.194^*^	0.114
Leather cotton per plant	257.685^**^	35.054^*^	42.290^**^	25.203^**^	7.905
Seed cotton per plant	1,220.353^**^	905.620^**^	220.025^**^	114.480^**^	40.851
Boll number per plant	39.406^**^	14.538^**^	7.186^**^	4.024^**^	1.25

**Notes.**

* Represents 0.05 level of significance.

** Represents 0.01 level of significance.

### Joint ANOVA of combining ability for yield traits across years

ANOVA results for combining ability (Table 4) indicated no significant differences between years for all yield traits. GCA differences among parents were significant or highly significant for all yield traits except cotton lint percentage and boll weight. SCA differences among combinations were highly significant for leather cotton per plant, seed cotton per plant, and boll number per plant. The interaction between year and parental GCA was significant or highly significant for all yield traits, and the interaction between year and SCA of combinations was significant for leather cotton per plant, seed cotton per plant, and boll number per plant.

**Table 4:** Joint ANOVA and mean square significance of yield trait fit in different years.

Nature (*i.e.*, properties of sth)	Vintages	Parentage GCA	Combined SCA	Year × parent GCA	Year × combination SCA	Inaccuracies
Seed index	0.223	4.800^*^	1.685	6.216^**^	1.358	1.755
Cotton lint percentage	1.950	61.221	13.845	65.753^*^	16.931	28.940
Boll weight	0.028	1.010	0.397	1.460^*^	0.419	0.540
Leather cotton per plant	29.852	43.070^**^	26.487^**^	40.769^**^	20.776^*^	10.008
Seed cotton per plant	111.789	206.181^**^	133.810^**^	167.421^**^	94.624^*^	51.473
Boll number per plant	2.428	5.824^**^	3.574^**^	6.724^**^	3.389^*^	1.617

**Notes.**

* Represents 0.05 level of significance.

** Represents 0.01 level of significance

### Analysis of genetic effects on yield traits in upland cotton

Combining ability analysis for yield traits across two years (Table 5) showed that all six yield traits were controlled by both additive and dominant genes. Given that the variance of GCA exceeded that of SCA for all six traits, it can be concluded that additive genetic effects predominantly controlled these traits, complemented by dominance effects. The genotype-environment interaction variance was substantial for all yield traits, indicating strong environmental influences. The highest broad-sense heritability was 70.263% for seed cotton per plant, and the lowest was 56.702% for boll weight.

**Table 5:** Variance of fit and heritability for different yield traits.

Nature (*i.e.*, properties of sth)	V_g_	V_s_	V_ge_	Broad-sense heritability H^2^ (%)
Seed index	4.800	1.685	7.574	60.546
Cotton lint percentage	61.221	13.845	82.684	60.712
Boll weight	1.010	0.397	1.880	56.702
Leather cotton per plant	43.070	26.487	61.544	67.642
Seed cotton per plant	206.181	133.810	262.045	70.263
Boll number per plant	5.824	3.574	10.114	63.250

### Correlation and stability analysis of GCA and SCA across years

Pearson correlation coefficients and stability analyses for parental GCA and SCA over two years (Table 6) showed no significant correlations between years for most yield traits except cotton lint percentage, suggesting inconsistent combining ability across years. Stability comparison coefficients for GCA of leather cotton per plant and seed cotton per plant were 51.372% and 55.187%, respectively. Stability coefficients for other traits ranged between 40% and 50%. Stability coefficients for SCA were relatively lower for cotton lint percentage (44.986%) and boll weight (48.645%), whereas coefficients for other traits ranged between 50% and 60%. Except for cotton lint percentage, whose SCA stability coefficient was lower than that of GCA, stability coefficients of SCA were generally higher than those of GCA, indicating that SCA exhibited greater stability than GCA for most traits across different years.

**Table 6:** Correlation and relative stability analysis of fitness of yield traits among different years.

Nature (*i.e.*, properties of sth)	GCA correlation coefficient Rg	SCA correlation coefficient Rs	GCA stability comparison coefficient Cg (%)	SCA stability comparison coefficient Cs (%)
Seed index	0.290	0.060	43.571	55.380
Cotton lint percentage	0.780 ^*^	0.330	48.215	44.986
Boll weight	0.400	0.230	40.892	48.645
Leather cotton per plant	0.060	0.300	51.372	56.042
Seed cotton per plant	0.280	0.360	55.187	58.577
Boll number per plant	0.370	0.290	46.413	51.330

**Notes.**

* Represents 0.05 level of significance.

### GCA analysis of yield traits for each parent across different years

**Figure 3:**
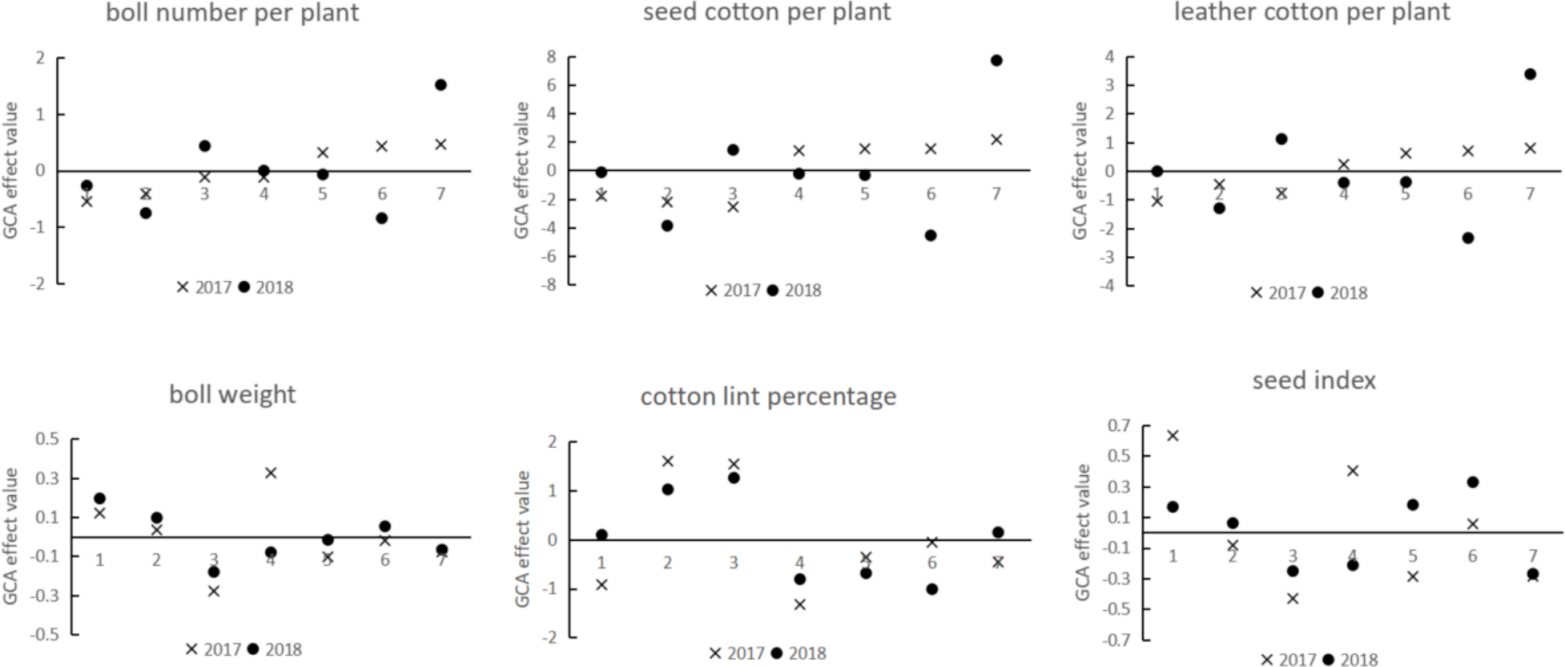
GCA effect values of parental yield traits in different years.

The GCA of seven upland cotton parents over two years was analysed. Results (Fig. 3) indicated that parents 1, 2, and 4 showed negative GCA effects for boll number per plant in both years, whereas parent 7 exhibited positive effects consistently. The other parents demonstrated inconsistent effects across the two years. For seed cotton per plant, parents 1 and 2 had consistently negative effects, parent 7 consistently showed a positive effect, and the remaining parents exhibited inconsistent effects. Similarly, for leather cotton per plant, parents 1 and 2 consistently displayed negative effects, parent 7 consistently showed a positive effect, and other parents had varying effects. Regarding boll weight, parents 1 and 2 demonstrated consistent positive effects, while parents 3, 5, and 7 showed consistent negative effects. For cotton lint percentage, parents 2 and 3 had consistently positive effects, parents 4, 5, and 6 had consistently negative effects, and others were inconsistent. Finally, for seed index, parents 1 and 6 consistently showed positive effects, parents 3 and 7 consistently had negative effects, and the remaining parents displayed varying effects.

### SCA analysis of yield traits for each hybrid combination across different years

**Figure 4:**
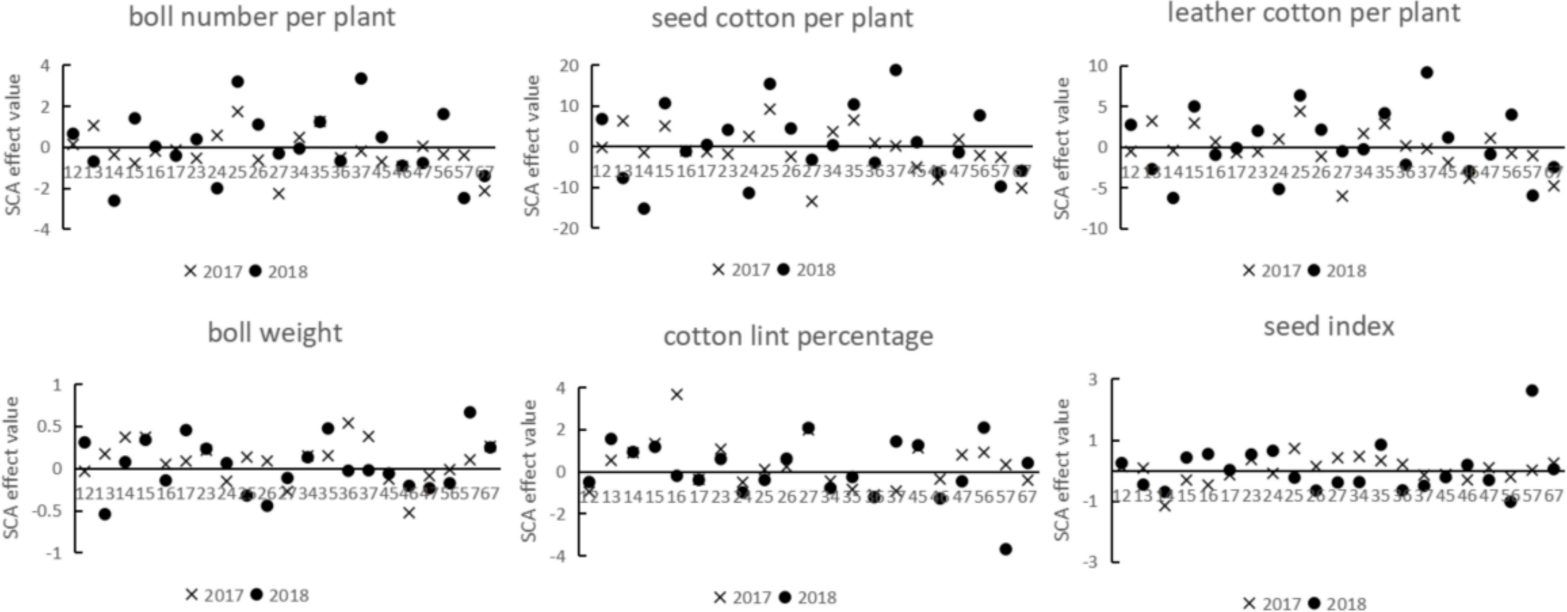
SCA effect values for combinations of yield traits in different years.

SCA of 21 combinations over two years (Fig. 4) revealed consistent positive effects for boll number per plant in combinations 12, 25, and 35, whereas combinations 14, 17, 27, 36, 46, 57, and 67 consistently showed negative effects. For seed cotton per plant, combinations 15, 25, 34, 35, and 37 consistently showed positive effects, whereas combinations 14, 16, 27, 46, 57, and 67 consistently exhibited negative effects. For leather cotton per plant, combinations 15, 25, and 35 consistently demonstrated positive effects, while combinations 14, 17, 27, 46, 57, and 67 showed consistent negative effects. For boll weight, combinations 14, 15, 17, 23, 34, 35, 57, and 67 consistently had positive effects, whereas combinations 27, 45, 46, 47, and 56 consistently exhibited negative effects. In terms of cotton lint percentage, combinations 13, 14, 15, 23, 26, 27, 45, and 56 consistently displayed positive effects, whereas combinations 12, 17, 24, 34, 35, 36, and 46 consistently exhibited negative effects. For seed index, combinations 12, 23, 35, and 67 consistently showed positive effects, while combinations 14, 17, 37, 45, and 56 consistently had negative effects. The remaining combinations demonstrated inconsistent effects across the two years.

### Stability analysis of combining ability for yield traits in parents and combinations across years

#### Stability analysis of GCA

**Figure 5:**
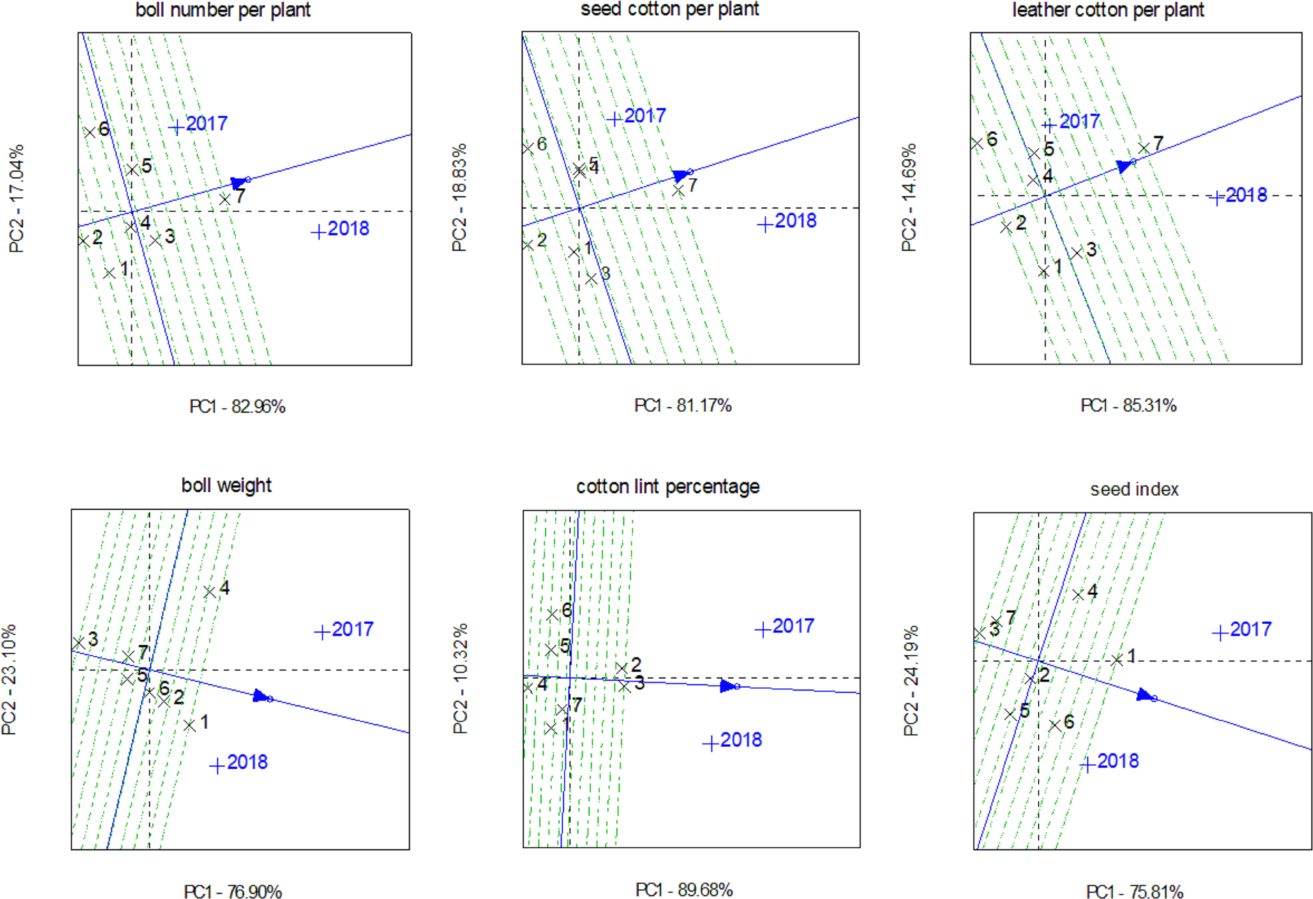
Analysis of GCA stability of parental yield traits in different years.

The stability of GCA for seven parents over two years (2017 and 2018) was analysed using GGE biplots (Fig. 5). Parents positioned right of the vertical axis performed above the overall mean, whereas those to the left performed below average. Parents positioned closer to the horizontal axis showed greater stability, while those further away exhibited instability.

 Parents 3, 5, and 7 had superior GCA for boll number per plant, with parent 7 being most stable and parents 3 and 5 less stable. For seed cotton per plant, parents 4, 5, and 7 exhibited higher GCA; parent 7 was again most stable, whereas parents 4 and 5 were comparatively unstable. Regarding leather cotton per plant, parents 3, 5, and 7 showed better GCA, with parent 7 displaying the highest stability. For boll weight, parents 1, 2, 4, and 6 had higher GCA; parents 2 and 6 showed greater stability, while parent 4 was the least stable. Parents 2 and 3 had better and stable GCA for cotton lint percentage. Parents 1, 4, and 6 exhibited better GCA for seed index, with parent 1 being most stable and parent 4 least stable.

#### Stability analysis of SCA

**Figure 6:**
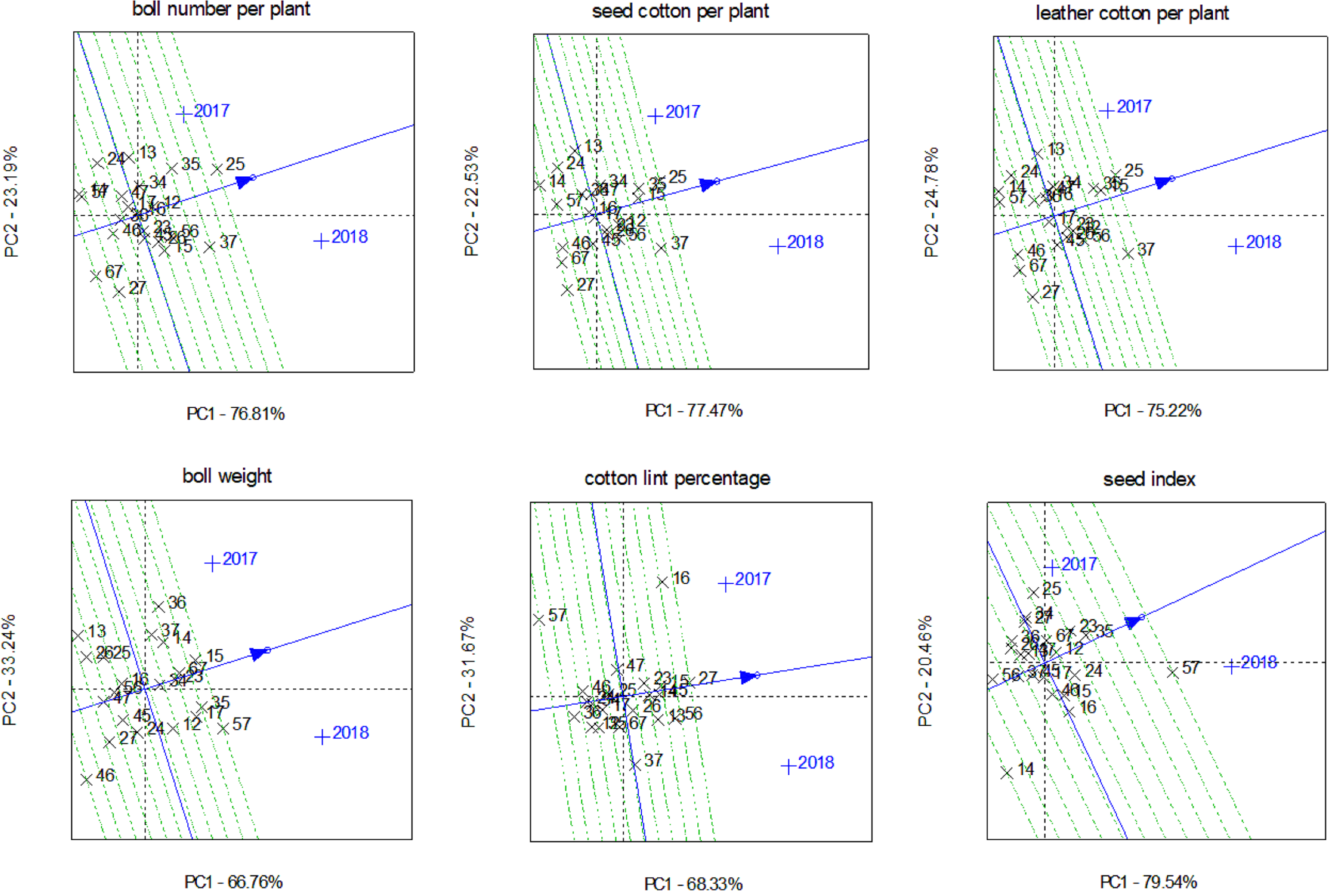
SCA stability analysis of yield traits of combinations in different years.

The stability of SCA for 21 combinations in both years was evaluated using GGE biplots (Fig. 6). Combinations on the right side of the vertical axis performed above the overall mean, while those on the left performed below average. Combinations nearer the horizontal axis were more stable; those further away showed less stability.

 For boll number per plant, combinations 12, 13, 15, 25, 26, 34, 35, 37, and 56 exhibited higher SCA; combination 12 had the best stability, while combinations 25, 26, 34, and 56 were relatively stable. For seed cotton per plant, combinations 12, 15, 23, 25, 26, 34, 35, 37, and 56 displayed better SCA; combination 15 was most stable, and combinations 12, 23, 25, and 35 showed relatively high stability. Regarding leather cotton per plant, combinations 12, 13, 15, 23, 25, 26, 34, 35, 37, and 56 had superior SCA, with combination 15 being most stable, followed by combinations 12, 23, 25, and 35. For boll weight, combinations 12, 14, 15, 17, 23, 34, 35, 36, 37, 57, and 67 demonstrated higher SCA; combinations 23 and 34 were most stable, while combinations 15 and 67 showed moderate stability. Combinations 13, 14, 15, 16, 23, 26, 27, 45, and 56 had better SCA for cotton lint percentage; combinations 14, 15, 27, and 45 were the most stable, followed by combinations 23 and 26. For seed index, combinations 12, 15, 23, 24, 25, 35, 57, and 67 showed higher SCA; combinations 12 and 35 had the highest stability, whereas combinations 23, 24, and 67 exhibited moderate stability.

## Discussion

### Genotype and environmental analysis of upland cotton yield traits

Cotton yield is influenced by multiple factors. Studies by Elsamman et al. (2024) and Galdi et al. (2022) indicated that the environment is a major determinant of cotton yield. In the present study, significant or highly significant yearly differences were found in all traits except seed index, supporting previous findings. Additionally, significant or highly significant differences appeared among genotypes and in genotype-year interactions for all traits. This indicates substantial genotype effects and genotype-by-environment interactions influencing yield traits, aligning with findings by Yehia (2022) and Askander (2020).

### Analysis of combining ability, correlation, and stability for yield traits across years

Environmental factors impacted traits across all parental lines, consistent with conclusions by Bhandari et al. (2021). For cotton combining ability, leather cotton per plant, seed cotton per plant, and boll number per plant exhibited significant or highly significant effects in parental GCA, hybrid SCA, and interactions involving year × parental GCA and year × hybrid SCA. In this study, correlations of SCA for six yield traits across years were generally weak. SCA showed greater stability across years compared with GCA. Traits such as leather cotton per plant, seed cotton per plant, and boll number per plant can thus serve as selection criteria for identifying superior hybrids.

Cotton yield traits were influenced by additive and dominant genetic effects. This finding is consistent with conclusions by Feng et al. (2022), Subalakhshmi et al. (2022), and Abdel-Aty et al. (2023), who reported yield traits predominantly controlled by additive effects, complemented by dominant effects. The current analysis also demonstrated significant environmental influence on all traits. Generalized heritability was highest for seed cotton per plant (70.263%) and lowest for boll weight (56.702%). These results can inform breeding programs aiming at improved cotton varieties.

### Selection of superior parents and hybrid combinations

Based on GGE biplot analysis, parent 7 (Xinluzao62) demonstrated the best overall GCA performance and stability among seven parents, making it suitable for future breeding programs. Among 21 hybrid combinations, combinations 12 (cm3 × a115-11), 15 (cm3 × Xinluzhong59), 23 (a115-11 ×Xinluzhong53), 25 (a115-11 ×Xinluzhong59), 26 (a115-11 ×Lu917), 34 (Xinluzhong53 ×Xinluzhong38), 35 (Xinluzhong53 ×Xinluzhong59), and 67 (Lu917 ×Xinluzao62) exhibited superior SCA performance and stability. These combinations can undergo further regional field evaluations. If future trials confirm the findings of this study, these hybrids can be recommended for broader regional adoption.

## Conclusions

Cotton yield traits differed significantly or highly significantly between years, except for the seed index, indicating a strong environmental influence. Significant or highly significant interactions occurred between genotype and genotype × year for all yield traits, suggesting substantial genotype differences and genotype-environment interactions. These findings align with those of Sharma et al. (2018). Cotton yield traits are primarily controlled by additive genetic effects, supplemented by dominant effects. Among these traits, the highest broad-sense heritability was observed for seed cotton per plant, and the lowest for boll weight. Traits including leather cotton per plant, seed cotton per plant, and boll number per plant were significant or highly significant in most hybrid combinations. Hybrid combinations exhibited greater stability in combining ability compared to parental lines across different years. In cotton breeding, seed cotton per plant could serve as the primary selection criterion, supported by leather cotton per plant and boll number per plant as auxiliary traits for selecting superior combinations. Eight combinations were identified using GGE biplots and combined analytical results: combinations 12 (cm3 × a115-11), 15 (cm3 × Xinluzhong59), 23 (a115-11 × Xinluzhong53), 25 (a115-11 × Xinluzhong59), 26 (a115-11 × Lu 917), 34 (Xinluzhong53 × Xinluzhong38), 35 (Xinluzhong53 × Xinluzhong59), and 67 (Lu917 × Xinluzao62). These combinations demonstrated stable yield traits and strong adaptability across environments. They can thus be utilized in breeding programs in different regions.

Future research should further examine the effects of additional environmental factors on cotton yield traits. Similar analytical methods can be applied to other crop species. Long-term regional trials on these eight combinations are required to confirm their broader applicability and to investigate underlying molecular mechanisms.

## Supplemental Information

10.7717/peerj.19716/supp-1 Supplemental Information 1 Raw data

10.7717/peerj.19716/supp-2 Supplemental Information 2 Climate raw data

## Additional Information and Declarations

### Data Availability

The following information was supplied regarding data availability: The data is available at Zenodo: - ma, . xiaoman . (2025). Climate data for 2021 and 2022 (Data set). Zenodo. https://doi.org/10.5281/zenodo.15761146
- ma, . xiaoman . (2025). Data on yield traits of upland cotton (Data set). Zenodo. https://doi.org/10.5281/zenodo.15761158
The original measurement values are available in the Supplementary Files.
